# Trajectories of psychotropic drug use patterns in psychiatric inpatients with acute depressive episodes of bipolar disorder: results from a pharmacovigilance program in German-speaking countries from 2007 to 2024

**DOI:** 10.1186/s40345-026-00435-9

**Published:** 2026-07-31

**Authors:** Johanna Seifert, Susanne Stübner, Dominik Dabbert, Waldemar Greil, Hannah B. Maier, Renate Grohmann, Sermin Toto

**Affiliations:** 1 Department of Psychiatry, Social Psychiatry and Psychotherapy, Hannover Medical School, Carl-Neuberg-Straße 1, 30625 Hannover, Germany; 2 Department of Psychiatry and Psychotherapy, University Hospital LMU, Munich, Germany; 3 Department of Forensic Psychiatry and Psychotherapy, Psychiatric University Hospital Zurich, Rheinau, Switzerland; 4 Department of Forensic Psychiatry and Psychotherapy, Klinik Bremen-Ost, Bremen, Germany; 5 Psychiatric Private Hospital, Sanatorium Kilchberg, Zurich, Switzerland; 6 Department of Psychiatry and Psychotherapy, School of Medicine & Health Sciences, Carl Von Ossietzky University of Oldenburg, Oldenburg, Germany

**Keywords:** Bipolar depression, Polypharmacy, Antipsychotic agents, Antidepressive agents, Mood stabilizers, Lithium, Pharmacoepidemiology

## Abstract

**Background:**

With limited evidence-based options, treatment of depressive episodes in bipolar disorder (BD-DE) remains challenging. This study examines the trajectories of psychotropic drug use and polypsychopharmacy in psychiatric inpatients with acute BD-DE.

**Methods:**

Using data from the German pharmacovigilance program “Arzneimittelsicherheit in der Psychiatrie” (AMSP), the pharmacological treatment of 1,902 psychiatric inpatients with BD-DE between 2007–2024 was analyzed. Temporal changes in drug use between early (T1: 2007–2010) and late (T2: 2021–2024) periods were calculated using prevalence ratios (PR) with 95% confidence intervals (CI) and Welch’s t-test.

**Results:**

Overall, 98.5% of inpatients with acute BD-DE were treated with psychotropic drugs. Antipsychotic drugs (APD) were the most used psychotropic drug group (76.2%), followed by antidepressant (ADD; 70.3%) and antiepileptic drugs (AED; 44.3%) during the entire study period. Over time, APD use increased from 70.1% in T1 to 80.7% in T2 (PR 1.15, CI 1.06–1.25), attributable to the increased use of second-generation APD (PR 1.19, CI 1.07–1.32), especially quetiapine and aripiprazole. ADD (T1: 78.1% vs T2: 67.6%; PR 0.87, CI 0.79–0.95) and AED (T1: 56.7% vs T2: 34.6%; PR 0.61, CI 0.51–0.73) use significantly declined, while lithium use remained stable (31.7% from 2007–2024). Although the mean number of psychotropic drugs per patient decreased (T1: 3.85 vs T2: 3.34, *p* < 0.001, *d*=−0.30), complex polypsychopharmacy consisting of ≥3 psychotropic drugs remained prevalent (66.1% of patients during the entire study period). Combination strategies involving APD, especially use of two APD, significantly increased (T1: 15.6% vs T2: 31.5%; PR 2.01, CI 1.51–2.69).

**Conclusion:**

Pharmacological treatment of BD-DE has shifted towards an increased use of second-generation APD while AED are less utilized. ADD use and complex polypsychopharmacy continue to be prevalent, indicating a persistent gap between guideline recommendations and real-world practice.

**Supplementary Information:**

The online version contains supplementary material available at 10.1186/s40345-026-00435-9.

## Introduction

Bipolar disorder (BD) is a severe mental disorder characterized by (hypo)manic and depressive episodes. When considering the entire bipolar spectrum, BD has a lifetime prevalence of up to 2.4% (Merikangas et al. 2007). In many patients, BD causes significant impairments in psychosocial functioning (Grande et al. 2016). It is also associated with a high risk of suicide attempts and completed suicide: 15–20% of bipolar patients die as a result of suicide (Dong et al. 2019). BD can be further classified as Bipolar I and Bipolar II. Bipolar I disorder is defined by mania (depression is typical but not required for diagnosis), whereas patients with Bipolar II have had at least one hypomanic and one major depressive episode (Grande et al. 2016). While the Diagnostic and Statistical Manual of Disease (5th Edition, DSM-V; American Psychiatric Association 2013) and the International Classification of Disease, 11th Edition (ICD-11; World Health Organization 2019) use this classification, the differentiation between Bipolar I and II is not found in the diagnostic criteria of the ICD-10 (World Health Organization 1992).

Treatment of patients with BD is per se complex because it must address different illness phases, including manic, depressive and mixed episodes as well as long-term maintenance. Patient and therapeutic needs can differ substantially between these phases, requiring regular monitoring and adjustment of treatment strategies over time. Manic episodes in BD (BD-ME) are generally clearly identifiable and, in many cases, require hospitalization. Pharmacological management can often be achieved with antipsychotic drugs (APD) and mood-stabilizing drugs – including lithium or various antiepileptic drugs (AED) – often in combination with each other. While (hypo-)mania prevails 1–10% of time, longitudinal studies indicate that patients with BD spend approximately 30–50% of their illness course in depressive states (Judd et al. 2002, 2003). Even in euthymic patients, subsyndromal symptoms of depression are common and significantly impact quality of life, daily functioning and illness course (Serafini et al. 2018). Moreover, depressive episodes following (hypo)mania may potentially be experienced as more burdensome because the pronounced shift from elevated to markedly reduced affectivity can amplify subjective distress (Kim et al. 2024). Effective pharmacological options for depressive episodes in BD (BD-DE) are unfortunately quite limited and patients with BD-DE are less likely to reach remission than patients with BD-ME (Hlastala et al. 1997). Treatment options include quetiapine, olanzapine, lurasidone, cariprazine and lamotrigine, although several of these are off-label depending on the specific region and their efficacy has yielded only small-to-medium effect sizes (Yildiz et al. 2023; Yalin et al. 2026). The use of antidepressant drugs (ADD) is not only “off-label” in most countries, it also carries the potential risk of inducing mania or rapid cycling (Barbuti et al. 2023).

Among available guidelines, the recommendations by the Canadian Network for Mood and Anxiety Treatments (CANMAT) and the International Society for Bipolar Disorders (ISBD) are widely used for the management of BD (Yatham et al. 2018). The CANMAT/ISBD guidelines recommend quetiapine, lurasidone (monotherapy or in combination with lithium or valproate), lithium, lamotrigine and adjunctive lamotrigine as first-line options for acute BD-DE. Second-line options include valproate, adjunctive SSRI or bupropion, cariprazine, olanzapine-fluoxetine combination, whereas aripiprazole, carbamazepine and olanzapine are among third-line options. In comparison, within the German S3 guidelines for BD (Bschor et al. 2020), quetiapine has the highest level of recommendation (level A, strong recommendation), followed by lurasidone in monotherapy or in combination with lithium or valproic acid (level B, moderate), while carbamazepine, lamotrigine and olanzapine receive weaker recommendation (level 0).

Directly reflecting the challenging treatment of BD, epidemiological studies have detected complex regimens of polypsychopharmacy (i. e., the use of two or more psychotropic drugs (Mojtabai and Olfson 2010) often including the use of three or more psychotropic drugs in ambulatory patients with BD-DE. Particularly the use of ADD or APD appears to bear a greater risk of polypsychopharmacy than AED or lithium (Goldberg et al. 2009). Despite the limited evidence supporting their use and their potential risks in BD-DE, ADD appear to be a common treatment option (Goldberg et al. 2009; Koistinaho et al. 2023; Tokumitsu et al. 2020), while the use of mood stabilizing drugs such as lithium – despite being guideline recommended and approved for BD – is concerningly low (Koistinaho et al. 2023).

Using data from the German pharmacovigilance program “Arzneimittelsicherheit in der Psychiatrie” (AMSP; German for “Drug Safety in Psychiatry”), two previous analyses have examined psychotropic drug use in hospitalized patients with acute BD-DE from 1993–2009 detecting high rates of ADD use in 81.3% of patients and frequent polypsychopharmacy of three or more psychotropic drugs (Haeberle et al. 2012; Greil et al. 2012). Further, there was a shift towards an increasing use of second-generation antipsychotics (SGA) and AED over the course of the study period, particularly quetiapine, lamotrigine and valproic acid (Greil et al. 2012). The present analysis is an extension of these findings, particularly in light of quetiapine’s approval for the treatment of BD-DE in 2008. Primary focus is the trajectory of psychotropic drug use and strategies over the course of 18 years from 2007 to 2024.

## Methods

### Study design and data source

“Arzneimittelsicherheit in der Psychiatrie” (AMSP; German for “Drug Safety in Psychiatry”) was established in 1993 and continues to operate as an ongoing pharmacovigilance program in Germany and Austria. Apart from monitoring severe adverse drug reactions, the program collects drug use data in psychiatric inpatients in hospitals participating in the AMSP project. The collected data includes drug use and dosage, diagnoses, age and sex of all patients currently in inpatient care in all project hospitals on two annual index days. The data used in this analysis stems from a total of 44 hospitals in Germany and Austria. A detailed report of AMSP’s methodology can be found elsewhere (Grohmann et al. 2004).

### Study population

This analysis includes all patients ≥ 18 years treated as inpatients at participating hospitals between 01.01.2007 and 31.12.2024 with a primary diagnosis (i.e., reason for hospitalization) of acute BD-DE. To maintain consistency of reporting throughout the study period, only project hospitals that submitted complete data sets between 2007 to 2024 were included. The present dataset has a two-year overlap with the earlier AMSP-based analyses (Haeberle et al. 2012; Greil et al. 2012), i.e., includes the same patient data; however, the current study greatly extends this observation window to reflect current trends.

### Diagnostic and clinical information

BD-DE was diagnosed by the physicians responsible for inpatient treatment according to the International Classification of Disease, 10th Edition (ICD-10). All patients treated with a primary diagnosis of ICD-10: F31.3, F31.4 or F31.5 between 2007 and 2024 were included, alongside data regarding age, sex and drug use.

### Classification of psychotropic drugs, polypsychopharmacy and analysis of drug use data

Only utilization rates of individual psychotropic drugs used in ≥ 2.5% of all patients treated for acute BD-DE from 2007–2024 are presented in this study. Psychotropic drugs were categorized as follows:

- Antidepressant drugs (ADD): selective serotonin reuptake inhibitors (SSRI), selective serotonin-norepinephrine reuptake inhibitors (SSNRI), tricyclic antidepressants (TCA), noradrenergic and specific serotonergic antidepressants (NaSSA), monoamine oxidase inhibitors (MAOI) and “other” ADD
- Antipsychotic drugs (APD): further classified as first-generation antipsychotics (FGA) with low or high potency (lp, hp respectively) and second-generation antipsychotics (SGA)
- Tranquilizing drugs (TRD; mainly benzodiazepines)
- Hypnotic drugs (HYPD; mainly so called “Z-drugs”, e.g., zolpidem or zopiclone)
- Mood stabilizers (including lithium) and antiepileptic drugs (AED; e.g., lamotrigine, valproic acid, carbamazepine, pregabalin)
- Antiparkinsonian drugs (mainly biperiden); used to treat treatment-emergent extrapyramidal symptoms

The term “psychotropic drug group” refers to broad therapeutic categories based on clinical indication (e.g., ADD, APD), whereas the expression “drug classes” refers to pharmacologically or mechanistically distinct subtypes (e.g., SSRI, SSNRI).

Polypsychopharmacy was defined as the concurrent use of two or more psychotropic drugs (Mojtabai and Olfson 2010). “Complex polypsychopharmacy” was defined as the use of three or more psychotropic drugs, according to a more recent systematic review (Kim et al. 2021). Various combination strategies involving different psychotropic drug groups (i.e., APD, ADD, AED, lithium, TRD, HYPD) were considered. Combinations of two and three psychotropic drug groups were analyzed. For the dual combinations, an additional analysis examining the use of different psychotropic drug classes of APD (i.e., SGA) and ADD (i.e., SSRI, SSNRI, NaSSA, TCA) was performed.

### Outcome measures

Main outcome measures included general use of different psychotropic drug groups, classes and individual psychotropic drugs, as well as the prevalence of polypsychopharmacy and different drug combination strategies. Temporal changes from the early to the late study period were assessed.

### Statistical methods

Statistical analyses and data visualization were performed using Microsoft Excel^©^ (Microsoft Corp., Redmond, WA, USA) and R version 4.5.3 (R Foundation for Statistical Computing, Vienna, Austria). For improved graphical visualization and interpretation, annual data was aggregated into four time intervals of 4–6 years: 2007–2010 (T1; N = 365), 2011–2014 (N = 559), 2015–2020 (N = 657) and 2021–2024 (T2; N = 321). To analyze changes in patient characteristics and drug use patterns, the first (T1) and last four-year interval (T2) of the study period were grouped and compared using prevalence ratios (PR). PR were calculated as the prevalence in T2 divided by the prevalence in T1 including their 95% confidence intervals (CI) and reflect differences in drug use prevalence rather than a risk (Tamhane et al. 2016). A PR greater than 1 indicates that the prevalence increased from T1 to T2, while a PR below 1 signifies a reduction.

We also applied a logistic regression model to assess overall temporal trends across all aggregated time intervals. Regression coefficients (β), standard errors (SE), odds ratios (OR) with 95% CI, and corresponding *p*-values were reported. These analyses address complementary aspects of the data, with PR providing pairwise comparisons between two time points (T1, T2) and the regression model assessing longitudinal trends.

For the analysis of polypsychopharmacy, the mean including the standard deviation (SD) and median number of psychotropic drugs were calculated. Mean drug use from T2 to T1 was compared using a Welch’s t-test. Cohen’s *d* was calculated as a measure of effect size (negligible *d* < 0.2, small *d* = 0.2, medium *d* = 0.5, large *d* = 0.8). The level of significance was set at *p* < 0.05.

### Ethical considerations

The Ethics Committee of the University of Munich and Hannover Medical School (Nr. 8100_BO_S_2018) have approved analyses using the AMSP database. This study adheres to the Declaration of Helsinki and its later amendments. As an observational program, AMSP does not interfere with the routine clinical treatment of patients under surveillance.

## Results

### General characteristics of the study population

The database identified a total of 1,902 patients who were treated for acute BD-DE within the study period from 2007–2024, among which 1,873 were treated with at least one psychotropic drug (98.5%). From 2007–2024, 61.7% of patients with BD-DE were female. Patients aged 31–60 years comprised the largest age group (57.2%). The median age was 54.5 years (range 19–96 years). Severe episodes without psychotic symptoms (ICD-10: F31.4; 69.3%) were more common than mild or moderate episodes (ICD-10: F31.3; 17.1%) and severe episodes with psychotic symptoms (ICD-10: F31.5; 13.6%). There were no significant differences in any of these patient characteristics between the first (2007–2010) and last (2021–2024) interval (Table 1).

**Table 1:** Characteristics (i.e., sex, age group, diagnosis) of the study population

	2007–2024		T1: 2007–2010		T2: 2021–2024		T2 vs T1
	N patients	% of all patients	N patients	% of all patients	N patients	% of all patients	PR (95% CI)
**Total**	1,902	100.0%	365	100.0%	321	100.0%	
**Sex**							
Female	1,174	61.7%	216	59.2%	203	63.2%	1.07 (0.95–1.20)
Male	728	38.3%	149	40.8%	118	36.8%	0.90 (0.75–1.09)
**Age group (in years)**							
≤ 30	133	7.0%	25	6.8%	22	6.9%	1.00 (0.58–1.74)
31–60	1,088	57.2%	214	58.6%	169	52.6%	0.90 (0.78–1.03)
> 60	681	35.8%	126	34.5%	130	40.5%	1.17 (0.97–1.42)
**Classification of bipolar depression (ICD-10)**							
Mild or moderate episode (F31.3)	326	17.1%	67	18.4%	49	15.3%	0.83 (0.59–1.16)
Severe episode without psychotic symptoms (F31.4)	1,318	69.3%	251	68.8%	236	73.5%	1.07 (0.97–1.18)
Severe episode with psychotic symptoms (F31.5)	258	13.6%	47	12.9%	36	11.2%	0.87 (0.58–1.31)

N: number (of); PR: prevalence ratio; CI: confidence interval; ICD-10: International classification of disease, 10th version

### Psychotropic drug use in bipolar depression from 2007–2024

Within the entire study period, APD were the most used psychotropic drug group (76.2% of patients with acute BD-DE), followed by ADD (70.3%), AED (44.3%), lithium (31.7%), TRD (27.7%) and HYPD (11.3%). APD use was largely due to SGA (66.0%), especially quetiapine (41.3%), whereas other SGA including aripiprazole (13.8%) and olanzapine (11.7%) were less common. Among FGA, lp FGA were used in 16.7% of patients, while hp FGA use was very low (3.6%). The utilization of ADD was highest for SSRI (26.4%), SSNRI (23.5%) and NaSSA (15.1%). TCA were used in the treatment of 7.4% and bupropion in 7.1% of patients. As the third most used psychotropic drug group, AED use was largely due to lamotrigine (17.9%) and valproic acid (20.9%; Table 2).

**Table 2:** Utilization of psychotropic drugs in patients with acute depressive episodes in bipolar disorder from 2007–2024 and comparison between early (T1: 2007–2010) and late (T2: 2021–2024) study periods

	2007–2024		T1: 2007–2010		T2: 2021–2024		T2 vs T1
	N	% of 1,902	N	% of 365	N	% of 321	PR (95% CI)
**Antipsychotic drugs (APD)**							
**Any APD**	1,449	76.2%	256	70.1%	259	80.7%	**1.15 (1.06–1.25)**
**SGA**	1,255	66.0%	225	61.6%	236	73.5%	**1.19 (1.07–1.32)**
Quetiapine	786	41.3%	129	35.3%	146	45.5%	**1.29 (1.07–1.55)**
Olanzapine	222	11.7%	42	11.5%	36	11.2%	0.97 (0.64–1.48)
Risperidone	151	7.9%	32	8.8%	22	6.9%	0.78 (0.46–1.32)
Aripiprazole	262	13.8%	27	7.4%	69	21.5%	**2.91 (1.91–4.42)**
**FGA lp**	318	16.7%	56	15.3%	55	17.1%	1.12 (0.79–1.57)
Pipamperone	115	6.0%	12	3.3%	34	10.6%	**3.22 (1.70–6.11)**
Promethazine	71	3.7%	15	4.1%	11	3.4%	0.83 (0.39–1.79)
Prothipendyl	80	4.2%	12	3.3%	18	5.6%	1.71 (0.83–3.49)
**FGA hp**	68	3.6%	14	3.8%	8	2.5%	0.65 (0.28–1.53)
**Antidepressant drugs (ADD)**							
**Any ADD**	1,337	70.3%	285	78.1%	217	67.6%	**0.87 (0.79–0.95)**
**SSRI**	502	26.4%	99	27.1%	92	28.7%	1.06 (0.83–1.34)
Escitalopram	156	8.2%	45	12.3%	24	7.5%	**0.61 (0.38–0.97)**
Citalopram	111	5.8%	23	6.3%	9	2.8%	**0.44 (0.21–0.95)**
Sertraline	198	10.4%	13	3.6%	56	17.4%	**4.90 (2.73–8.79)**
**SSNRI**	447	23.5%	96	26.3%	72	22.4%	0.85 (0.65–1.11)
Venlafaxine	325	17.1%	72	19.7%	46	14.3%	0.73 (0.52–1.02)
Duloxetine	93	4.9%	19	5.2%	13	4.0%	0.78 (0.39–1.55)
**NaSSA**	288	15.1%	76	20.8%	39	12.1%	**0.58 (0.41–0.83)**
Mirtazapine	286	15.0%	74	20.3%	39	12.1%	**0.60 (0.42–0.86)**
**TCA**	140	7.4%	44	12.1%	13	4.0%	**0.34 (0.18–0.61)**
Amitriptyline	47	2.5%	9	2.5%	6	1.9%	0.76 (0.27–2.11)
**MAOI**	51	2.7%	7	1.9%	6	1.9%	0.97 (0.33–2.87)
**Other ADD**	263	13.8%	55	15.1%	46	14.3%	0.95 (0.66–1.37)
Trazodone	58	3.0%	21	5.8%	10	3.1%	0.54 (0.26–1.13)
Bupropion	135	7.1%	21	5.8%	29	9.0%	1.57 (0.91–2.70)
Agomelatine	60	3.2%	8	2.2%	7	2.2%	0.99 (0.36–2.71)
**Antiepileptic drugs (AED)**							
**Any AED**	843	44.3%	207	56.7%	111	34.6%	**0.61 (0.51–0.73)**
Pregabalin	92	4.8%	19	5.2%	20	6.2%	1.20 (0.65–2.20)
Lamotrigine	341	17.9%	70	19.2%	55	17.1%	0.89 (0.65–1.23)
Carbamazepine	53	2.8%	26	7.1%	4	1.2%	**0.17 (0.06–0.50)**
Valproic acid	397	20.9%	111	30.4%	42	13.1%	**0.43 (0.31–0.59)**
**Lithium**							
**Any lithium salt**	602	31.7%	119	32.6%	100	31.2%	0.96 (0.77–1.19)
**Tranquilizing drugs (TRD)**							
**Any TRD**	526	27.7%	128	35.1%	73	22.7%	**0.65 (0.51–0.83)**
Lorazepam	392	20.6%	84	23.0%	54	16.8%	**0.73 (0.54–0.99)**
Diazepam	78	4.1%	27	7.4%	11	3.4%	**0.46 (0.23–0.92)**
**Hypnotic drugs (HYPD)**							
**Any HYPD**	215	11.3%	66	18.1%	25	7.8%	0.43 (0.28–0.67)
Zopiclone	131	6.9%	49	13.4%	9	2.8%	**0.21 (0.10–0.42)**
Zolpidem	50	2.6%	6	1.6%	8	2.5%	1.52 (0.53–4.32)
**Antiparkinsonian drugs**							
**Any antiparkinsonian drug**	74	3.9%	13	3.6%	13	4.0%	1.14 (0.53–2.42)

PR printed in bold indicate a significant change. N: number (of); PR: prevalence ratio; CI: confidence interval; SSRI: selective serotonin reuptake inhibitor; SSNRI: selective serotonin-norepinephrine reuptake inhibitor; TCA: tricyclic antidepressant; NaSSA: noradrenergic and specific serotonergic antidepressant; MAOI: monoamine oxidase inhibitor; FGA: first-generation antipsychotic drug; lp: low potency; hp: high potency; SGA: second-generation antipsychotic drug

### Trajectories of psychotropic drug use in bipolar depression over the study period

**Fig. 1:**
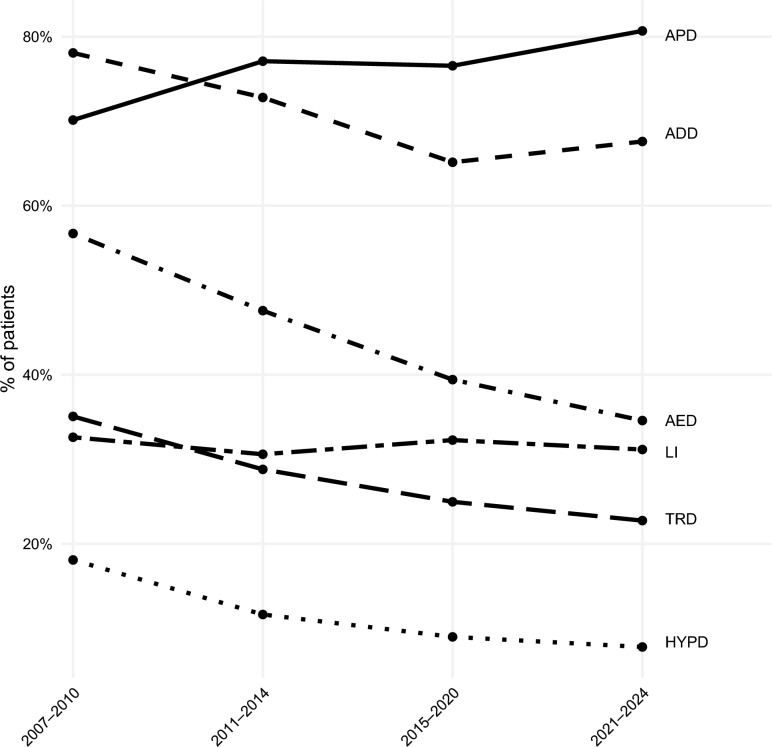
Percent of patients with acute depressive episodes in bipolar disorder treated with different psychotropic drug groups over the duration of the study period (2007–2024). For improved visualization, the data are aggregated into intervals of 4–6 years across the 18-year study period. From 2007–2010 to 2021–2024, APD use increased significantly (*p* = 0.004), while the use of ADD (*p* < 0.001), AED (*p* < 0.001), TRD (*p* < 0.001) and HYPD (*p* < 0.001) declined. Lithium utilization remained stable over time (*p* > 0.05). APD: antipsychotic drug, ADD: antidepressant drug, AED: antiepileptic drug, LI: lithium, TRD: tranquilizing drug, HYPD: hypnotic drug

Logistic regression analyses revealed significant temporal changes in the utilization of several psychotropic drug groups over the course of the study period (Fig. 1). The use of APD increased significantly over time (β = 0.160, SE = 0.055, OR = 1.17, CI 1.05–1.30, *p* = 0.004). In contrast, the use of ADD (β = −0.208, SE = 0.052, OR = 0.81, CI 0.73–0.90, *p* < 0.001), AED (β = −0.312, SE = 0.048, OR = 0.73, CI 0.67–0.80, *p* < 0.001), TRD (β = −0.207, SE = 0.052, OR = 0.81, CI 0.73–0.90, *p* < 0.001) and HYPD (β = −0.342, SE = 0.075, OR = 0.71, CI 0.61–0.82, *p* < 0.001) decreased significantly. Lithium use remained stable over time (β = −0.007, SE = 0.050, OR = 0.99, CI 0.90–1.09, *p* = 0.896).

The analysis of drug use within the different psychotropic drug groups when comparing the first period (2007–2010, T1) with the later (2021–2024, T2) further revealed several significant differences (Table 2):

***Antipsychotic drugs*** – The use of APD significantly increased from 70.1% in T1 to 80.7% in T2 (PR 1.15, CI 1.06–1.25), largely due to the rising utilization of SGA from 61.6 to 73.5% (PR 1.19, CI 1.07–1.32). The use of aripiprazole rose from 7.4% in T1 to 21.5% of patients in T2 (PR 2.91, CI 1.91–4.42), while quetiapine use showed a more modest increase from 35.3 to 45.5% (PR 1.29, CI 1.07–1.55). Olanzapine and risperidone did not show any temporal trends. While the use of lp FGA remained stable, pipamperone was significantly more utilized in T2 (PR 3.22, CI 1.70–6.11).

***Antidepressant drugs*** – ADD use showed a significant decline over time from 78.1% in T1 to 67.6% in T2 (PR 0.87, CI 0.79–0.95). When considering the different ADD classes, significant changes in drug utilization were found for NaSSA (T1: 20.8% vs T2: 12.1%; PR 0.58, CI 0.41–0.83) and TCA (T1: 12.1% vs T2: 4.0%; PR 0.34, CI 0.18–0.61). Several significant shifts in SSRI choice were detected: While both citalopram and escitalopram use decreased, sertraline use increased by nearly a 5-fold (T1: 3.6% vs T2: 17.4%; PR 4.90, CI 2.73–8.79).

***Antiepileptic drugs*** – AED use declined significantly from 56.7% in T1 to 34.6% of patients in T2 (PR 0.61, CI 0.51–0.73). This shift was mostly accounted for by the decreasing use of valproic acid. While it was the most used AED during T1 (30.4%), its use declined by more than half by T2 (13.1%; PR 0.43, CI 0.31–0.59). The use of carbamazepine also fell from 7.1% in T1 to 1.2% in T2 (PR 0.17, CI 0.06–0.50). Lamotrigine use remained stable, although it represented the most used AED in T2.

***Tranquilizing and hypnotic drugs*** – The use of both TRD and HYPD decreased significantly over time. While TRD were used in 35.1% of patients in T1, only 22.7% received them in T2 (PR 0.65, CI 0.51–0.83). HYPD use fell from 18.1% in T1 to 7.8% in T2 (PR 0.43, 0.28–0.67).

### Polypsychopharmacy in patients with bipolar depression

**Fig. 2:**
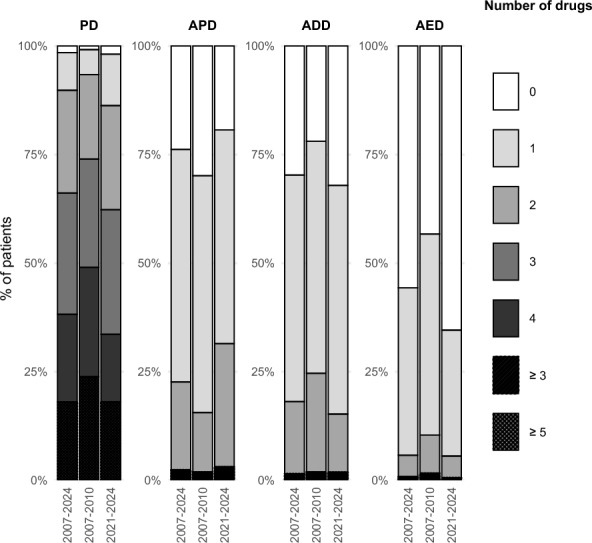
Distribution of the number of psychotropic drugs (PD), antipsychotic drugs (APD), antidepressant drugs (ADD) and antiepileptic drugs (AED) per patient in 2007–2024, 2007–2010 (T1) and 2021–2024 (T2) in patients with acute depressive episodes in bipolar disorder. Bars represent the percentage of patients receiving a given number of drugs per category. Categories with higher levels of polypsychopharmacy (≥ 3 or ≥ 5 drugs) are highlighted by patterned fills. Overall, the mean number of psychotropic drugs per patient decreased over time, whereas APD use increased and ADD use showed a slight decrease

Figure 2 shows the distribution of the number of psychotropic drugs, APD, ADD and AED per patient across all study periods. Overall, the number of psychotropic drugs showed a modest decrease from T1 (3.85 ± 1.69) to T2 (3.34 ± 1.75; t = −3.79, *p* < 0.001, *d* = −0.30), reflecting a shift from higher levels of polypsychopharmacy towards fewer drugs. With three psychotropic drugs per patient in all time periods, the median drug use did not change over time. Patients in the later time period were more likely to be treated with one psychotropic drug (PR 2.06, CI 1.23–3.43) and less likely to receive four psychotropic drugs (PR 0.62, CI 0.45–0.84). For APD, the mean number slightly increased from 1.05 ± 0.94 APD per patient in T1 to 1.22 ± 0.83 in T2 (t = 2.68, *p* = 0.008, *d* = 0.20). There was a notable rise in patients receiving two APD (PR 2.07, CI 1.52–2.82) and a decrease in those with no APD (PR 0.65, CI 0.49–0.85). Among ADD, the mean number of ADD decreased from 1.05 ± 0.91 (T1) to 0.89 ± 0.88 (T2) with small effect size (t = −2.36, *p* = 0.018, *d* = −0.18). This was particularly apparent for the use of two ADD (PR 0.59, CI 0.42–0.82), while treatment without any ADD increased (PR 1.48, CI 1.14–1.88). The percent of patients treated with one AED decreased from T1 to T2 (PR 0.63, CI 0.51–0.77), while significantly more patients did not receive any AED (PR 1.51, CI 1.31–1.74). The mean number of AED per patient significantly decreased from 0.69 ± 0.72 (T1) to 0.41 ± 0.62 (T2; t = −5.58, *p* < 0.001, *d* = −0.42) with small to moderate effect size (Suppl. Table 1).

### Use of different combinations of psychotropic drug groups

Overall, the most common combination of two psychotropic drug groups was the use of APD + ADD (51.9% of patients with BD-DE) followed by APD + AED (32.9%) and ADD + AED (32.2%). Combination treatment patterns changed substantially over time. The proportion of patients receiving APD + APD increased significantly from 15.6% in T1 to 31.5% in T2 (PR 2.01, 95% CI 1.51–2.69) and represents the only dual combination which increased in use. In contrast, combinations involving AED, TRD and HYPD showed a consistent decrease in almost all pairings. Notably, ADD-based combinations declined significantly for ADD (T1: 24.7% vs T2: 15.0%; PR 0.61, CI 0.44–0.83), AED (T1: 44.7% vs T2: 25.5%; PR 0.57, CI 0.46–0.71), TRD and HYPD from T1 to T2. A similar trend was found for AED-based combinations. Among combinations with lithium, the concomitant use of ADD, APD and TRD did not show significant changes over time, while the use of lithium with AED and HYPD decreased (Table 3).

**Table 3:** Utilization of dual combinations of psychotropic drug groups in patients with acute depressive episodes in bipolar disorder from 2007–2024 and comparison between early (T1: 2007–2010) and late (T2: 2021–2024) study periods

		2007–2024		T1: 2007–2010		T2: 2021–2024		T2 vs T1
		N	% of 1,902	N	% of 365	N	% of 321	PR (95% CI)
**Combinations with antipsychotic drugs (APD)**								
Any APD +	APD	431	22.7%	57	15.6%	101	31.5%	**2.01 (1.51–2.69)**
	ADD	987	51.9%	195	53.4%	166	51.7%	0.97 (0.84–1.12)
	AED	626	32.9%	140	38.4%	88	27.4%	**0.71 (0.57–0.89)**
	LI	440	23.1%	82	22.5%	75	23.4%	1.04 (0.79–1.37)
	TRD	434	22.8%	95	26.0%	65	20.2%	0.78 (0.59–1.03)
	HYPD	168	8.8%	47	12.9%	20	6.2%	**0.48 (0.29–0.80)**
**Combinations with antidepressant drugs (ADD)**								
Any ADD +	ADD	345	18.1%	90	24.7%	48	15.0%	**0.61 (0.44–0.83)**
	AED	613	32.2%	163	44.7%	82	25.5%	**0.57 (0.46–0.71)**
	LI	437	23.0%	94	25.8%	75	23.4%	0.91 (0.70–1.18)
	TRD	376	19.8%	103	28.2%	49	15.3%	**0.54 (0.40–0.73)**
	HYPD	166	8.7%	53	14.5%	19	5.9%	**0.41 (0.25–0.67)**
**Combinations with antiepileptic drugs (AED)**								
Any AED +	AED	110	5.8%	38	10.4%	18	5.6%	**0.54 (0.31–0.92)**
	LI	166	8.7%	47	12.9%	23	7.2%	**0.56 (0.35–0.90)**
	TRD	250	13.1%	73	20.0%	30	9.3%	**0.47 (0.31–0.70)**
	HYPD	89	4.7%	33	9.0%	9	2.8%	**0.31 (0.15–0.64)**
**Combinations with lithium (LI)**								
LI +	TRD	150	7.9%	39	10.7%	21	6.5%	0.61 (0.37–1.02)
	HYPD	74	3.9%	25	6.8%	5	1.6%	**0.23 (0.09–0.59)**

PR printed in bold indicate a significant change. N: number (of); PR: prevalence ratio; CI: confidence interval; HYPD: hypnotic drug; TRD: tranquilizing drug

Similarly, the use of psychotropic drug class in combination with different psychotropic drug groups shifted over time. Combination strategies involving SGA + APD significantly increased from 15.3% in T1 to 29.9% in T2 (PR 1.95, CI 1.45–2.61), while the combined use of SGA + AED decreased (T1: 33.4% vs T2: 24.0%; PR 0.72, CI 0.56–0.91). Among the three ADD classes SSNRI, NaSSA and TCA, all combinations with AED, TRD and HYPD significantly decreased. All combinations of SSRI with different psychotropic drug groups remained unchanged over time (Suppl. Table 2).

The three most used triple combinations from 2007–2024 were centered around the use of at least one APD: APD + ADD + AED (22.9%), APD + ADD + lithium (16.2%) and APD + ADD + TRD (16.1%). The use of APD + APD + ADD was the only triple combination with a significant increase over time from 10.1% in T1 to 19.3% in T2 (PR 1.91, CI 1.30–2.78). All other triple combinations either remained stable or showed a decline in use. Several combinations involving AED significantly decreased including APD + ADD + AED (T1: 29.3% vs T2: 19.3%; PR 0.66, CI 0.50–0.87) and ADD + ADD + AED (T1: 15.9% vs T2: 7.2%; PR 0.45, CI 0.28–0.71). Various combinations containing lithium significantly decreased including ADD + ADD + lithium (T1: 9.9% vs T2: 5.6%; PR 0.57, CI 0.33–0.98) and ADD + AED + lithium (T1: 9.9% vs T2: 5.0%; PR 0.51, CI 0.29–0.89). Almost all triple combinations containing TRD and HYPD significantly declined in use (Table 4).

**Table 4:** Utilization of triple combinations of psychotropic drug groups in patients with acute depressive episodes in bipolar disorder from 2007–2024 and comparison between early (T1: 2007–2010) and late (T2: 2021–2024) study periods

		2007–2024		T1: 2007–2010		T2: 2021–2024		T2 vs T1
		N	% of 1902	N	% of 365	N	% of 321	PR (95% CI)
APD + APD +	APD	46	2.4%	7	1.9%	10	3.1%	1.62 (0.63–4.22)
	ADD	274	14.4%	37	10.1%	62	19.3%	**1.91 (1.30–2.78)**
	AED	203	10.7%	35	9.6%	41	12.8%	1.33 (0.87–2.04)
	LI	112	5.9%	18	4.9%	24	7.5%	1.52 (0.84–2.74)
	TRD	138	7.3%	25	6.8%	33	10.3%	1.50 (0.91–2.47)
	HYPD	55	2.9%	14	3.8%	9	2.8%	0.73 (0.32–1.67)
APD + ADD +	ADD	249	13.1%	58	15.9%	39	12.1%	0.76 (0.52–1.11)
	AED	435	22.9%	107	29.3%	62	19.3%	**0.66 (0.50–0.87)**
	LI	308	16.2%	65	17.8%	51	15.9%	0.89 (0.64–1.25)
	TRD	306	16.1%	76	20.8%	42	13.1%	**0.63 (0.44–0.89)**
	HYPD	127	6.7%	36	9.9%	16	5.0%	**0.51 (0.29–0.89)**
ADD + ADD +	ADD	29	1.5%	7	1.9%	5	1.6%	0.81 (0.26–2.53)
	AED	175	9.2%	58	15.9%	23	7.2%	**0.45 (0.28–0.71)**
	LI	118	6.2%	36	9.9%	18	5.6%	**0.57 (0.33–0.98)**
	TRD	111	5.8%	32	8.8%	16	5.0%	0.57 (0.32–1.02)
	HYPD	47	2.5%	19	5.2%	5	1.6%	**0.30 (0.11–0.79)**
ADD + AED +	AED	83	4.4%	27	7.4%	15	4.7%	0.63 (0.34–1.17)
	LI	126	6.6%	36	9.9%	16	5.0%	**0.51 (0.29–0.89)**
	TRD	178	9.4%	60	16.4%	20	6.2%	**0.38 (0.23–0.61)**
	HYPD	71	3.7%	29	7.9%	9	2.8%	**0.35 (0.17–0.73)**
APD + AED +	AED	88	4.6%	30	8.2%	15	4.7%	0.57 (0.31–1.04)
	LI	130	6.8%	33	9.0%	20	6.2%	0.69 (0.40–1.18)
	TRD	201	10.6%	49	13.4%	28	8.7%	0.65 (0.42–1.01)
	HYPD	65	3.4%	20	5.5%	7	2.2%	**0.40 (0.17–0.93)**
AED + AED +	AED	16	0.8%	6	1.6%	2	0.6%	0.38 (0.08–1.86)
	LI	20	1.1%	8	2.2%	2	0.6%	0.28 (0.06–1.33)
	TRD	34	1.8%	12	3.3%	8	2.5%	0.76 (0.31–1.83)
	HYPD	9	0.5%	4	1.1%	1	0.3%	0.28 (0.03–2.53)
LI + APD +	TRD	121	6.4%	28	7.7%	17	5.3%	0.69 (0.39–1.24)
	HYPD	59	3.1%	18	4.9%	3	0.9%	**0.19 (0.06–0.64)**
LI + ADD +	TRD	108	5.7%	30	8.2%	14	4.4%	**0.53 (0.29–0.98)**
	HYPD	59	3.1%	21	5.8%	4	1.2%	**0.22 (0.08–0.62)**
LI + AED +	TRD	49	2.6%	19	5.2%	4	1.2%	**0.24 (0.08–0.70)**
	HYPD	21	1.1%	9	2.5%	2	0.6%	0.25 (0.05–1.16)

PR printed in bold indicate a significant change. ADD: antidepressant drug; APD: antipsychotic drug; HYPD: hypnotic drug; TRD: tranquilizing drug; AED: antiepileptic drug; N: number (of); PR: prevalence ratio; CI: confidence interval

## Discussion

This study analyzed the pharmacological treatment of 1,902 patients with acute BD-DE within the inpatient psychiatric setting over an 18-year period. While ADD were the most used psychotropic drug group in 2007–2010, their use decreased significantly by 2021–2024, at which point APD were most common. APD use – specifically of SGA – showed a significant increase over time, mainly driven by aripiprazole and quetiapine. AED, TRD and HYPD use declined significantly, while lithium use remained stable. Although there was a modest reduction in polypsychopharmacy from 2007–2010 to 2021–2024, combination strategies of different psychotropic drugs remained the mainstay of treatment with approximately two-thirds of patients receiving three or more psychotropic drugs simultaneously. When interpreting the results of this analysis, it is important to bear in mind that the observed drug use may in part reflect the continuation of pre-existing treatment rather than initiation for acute BD-DE. In the following, the results of the present study will be discussed acknowledging previous findings using AMSP data to assess the treatment of hospitalized patients with acute BD-DE from 1993 to 2009 (Greil et al. 2012; Haeberle et al. 2012).

### Increasing use of second-generation antipsychotic drugs

While Greil et al. (2012) reported a rising use of SGA in hospitalized patients with BD-DE, the present analysis indicates that this trend has not only persisted but further intensified in recent years, aligning with growing evidence of their efficacy in BD-DE. Meta-analyses have revealed that several SGA including quetiapine, lurasidone, cariprazine and olanzapine (especially in combination with fluoxetine) showed the highest evidence of effectively reducing symptoms of acute BD-DE, albeit at only a moderate level of evidence (Yildiz et al. 2023; Yalin et al. 2026). As a result, SGA are increasingly recommended as first-line treatment options for patients with BD-DE by current international treatment guidelines (Bschor et al. 2020; Yatham et al. 2018).

Consistent with this evidence, we found a substantial increase in the use of quetiapine as an in-label treatment option for BD-DE in Germany. The German S3 guideline for BD specifically proposes quetiapine for the treatment of BD-DE with the highest recommendation grade and it is the only drug to achieve this level of recommendation within this indication (Bschor et al. 2020). Quetiapine’s efficacy in BD is not limited to BD-DE; it is also effective in the treatment of BD-ME (Kishi et al. 2022) and relapse prevention of both BD-DE and BD-ME (Miura et al. 2014). We also found an increasing use of aripiprazole, which is more difficult to interpret. Although established in the treatment and maintenance therapy of BD-ME (Muneer 2016), its usefulness in acute BD-DE is questionable (Yildiz et al. 2023; Yalin et al. 2026). Also, though proven one of the most efficacious treatment option in BD-DE (Yildiz et al. 2023; Yalin et al. 2026), the decreasing use of olanzapine in this study may largely be the result of valid concerns pertaining to metabolic adverse drug reactions, such as weight gain and diabetes, especially in long-term use (Burschinski et al. 2023).

### Declining use of antidepressant drugs

Representing the most-used psychotropic drug class in the first study period (2007–2010), ADD use declined from 78.1 to 67.6% by 2021–2024. While this indicates a significant reduction, ADD use remained consistently high in BD-DE, quite like the findings by Greil et al. (2012). The reduction of ADD use in the present analysis aligns with growing awareness regarding not only their risks when used in BD-DE, but also their limited evidence of efficacy. Initial reports suggesting that ADD may induce mania stem from the 1970’s (Wehr and Goodwin 1987, 1979). TCA in particular have been associated with a higher risk for switch to mania than other ADD (Koszewska and Rybakowski 2009), whereas bupropion has been deemed a lower risk option (Manwani et al. 2006). A recent meta-analysis investigated whether ADD increase the risk of BD-ME (Oliva et al. 2025): The authors found no evidence that ADD – including TCA – induce mania more often than placebo, detecting only an insignificant signal for venlafaxine and therefore concluded ADD may be safe for use in acute BD-DE. However, the authors also note that the overall certainty of evidence was rated low due to small sample sizes, heterogeneity in outcomes and short trial durations (Oliva et al. 2025). Further, the evidence for ADD efficacy in BD-DE should be carefully considered: Apart from fluoxetine (low- to very low-quality evidence), recent meta-analyses were unable to determine any clinically relevant efficacy for all ADD examined (e.g., venlafaxine, sertraline, bupropion; Yildiz et al. 2023; Yalin et al. 2026). In so, the limited efficacy of ADD in acute BD-DE is consistent with evidence from unipolar depression, where ADD also only show modest effect sizes (Cipriani et al. 2018). While both the current German S3 and the CANMAT/ISBD guidelines are restrictive, the S3 guideline is neutral stating that current evidence doesn’t allow clear recommendations pertaining to the use of ADD in BD-DE (Bschor et al. 2020); in contrast, CANMAT/ISBD is more hierarchical indicating ADD are second-line only and clearly discourages monotherapy in Bipolar I disorder (Yatham et al. 2018). When ADD are considered, both guidelines suggest that SSRI and bupropion appear more favorable in comparison to TCA and SSNRI in regard to their risk of treatment-emergent mania (Bschor et al. 2020; Yatham et al. 2018). Despite these controversies and their lack of high-grade recommendation in contemporary treatment guidelines (Bschor et al. 2020; Yatham et al. 2018), other studies have also detected high rates of ADD use spanning from 41 to 66% in ambulatory patients with BD-DE (Tokumitsu et al. 2020; Koistinaho et al. 2023; Jain et al. 2022). Unawareness of current guideline recommendations may be one reason for the continuous high ADD use in BD-DE; however, urgent clinical necessity and general lack of highly effective treatment options for BD-DE may contribute. Also, anxiety disorders are among the most prevalent psychiatric comorbidities in patients with bipolar disorder (Léda-Rêgo et al. 2024) and may therefore have influenced drug selection as several ADD are regarded first-line pharmacological treatment options for different types of anxiety disorders.

### Declining use of mood-stabilizing antiepileptic drugs

AED use substantially decreased from 2007–2024 with a particularly prominent decline in valproic acid, which had previously shown an increasing use from 1993–2009 (Greil et al. 2012). While valproic acid was the most used AED in 2007–2010, its use not only declined but it was also surpassed by lamotrigine. Among AED with mood-stabilizing properties, lamotrigine was the only AED with stable utilization over the study period. The efficacy of lamotrigine is not limited to the relapse prevention of BD-DE, it has also proven efficacious in the treatment of acute BD-DE (Haenen et al. 2024) with moderate efficacy (Yildiz et al. 2023; Yalin et al. 2026). Despite this, lamotrigine only received a weak level of recommendation in the German S3 guideline (Bschor et al. 2020), unlike its first-line ranking in the CANMAT/ISBD guidelines (Yatham et al. 2018). Both valproic acid and carbamazepine are not associated with significant antidepressant effects in BD-DE (Yildiz et al. 2023; Yalin et al. 2026), they are, however, useful in relapse prevention of BD-ME (Yee et al. 2021).

The use of AED in BD carries one major disadvantage: Unlike quetiapine’s multifaceted efficacy in all phases of the treatment of BD, the efficacy of lamotrigine, carbamazepine and valproic acid is limited to either treatment/prevention BD-ME or treatment/prevention of BD-DE. Depending on individual patient needs, patients may require both relapse prevention of BD-ME and BD-DE, therefore necessitating the use of multiple psychotropic drugs. Next to drug safety concerns of carbamazepine and valproic acid regarding teratogenicity – especially pertaining to valproic acid (Christensen et al. 2024; Alsdorf and Wyszynski 2005) –, adverse drug reactions (LoPinto-Khoury 2022) and the risk of drug-drug-interactions (Perucca 2006) may have additionally contributed to the decreasing use of both of these AED.

### Stability of lithium use

While Greil et al. (2012) previously described a decrease from 44.8 to 34.4% of lithium use in patients with BD-DE from 1993–2009, this trend did not continue. Used in slightly under one third of patients with BD-DE, lithium – unlike all other psychotropic drug groups – was void of relevant temporal changes from T1 to T2. While at first glance it can be deemed a success that its use did not decline despite the increasing APD use in this analysis, it remains unfortunate that lithium is not used more frequently in BD (Bindel and Seifert 2025). Despite limited evidence of usefulness in acute BD-DE (Yildiz et al. 2023; Yalin et al. 2026; Rakofsky et al. 2022), lithium remains a first-line treatment recommendation in the CANMAT/ISBD guideline. This is due to its robust effects on relapse prevention as well as its unique anti-suicidal properties, emphasizing lithium’s overall long-term benefits rather than findings from acute-phase trials of limited duration (Yatham et al. 2018). The German S3 guideline does not share this recommendation for acute BD-DE, however, it does continue to recommend lithium as the gold standard in maintenance therapy (Bschor et al. 2020). Lithium has proven superior as monotherapy in the maintenance treatment of BD compared to other psychotropic drugs including quetiapine, olanzapine, lamotrigine and valproic acid (Kessing et al. 2018).

### Polypsychopharmacy in the treatment of bipolar depression

Another central finding of the present study is the high prevalence of complex polypsychopharmacy (i.e., use of ≥ 3 psychotropic drugs) among inpatients with acute BD-DE. Most patients (66.1%) in the present analysis received three or more psychotropic drugs from 2007 to 2024. We did, however, detect a modest decrease in the average number of psychotropic drugs used per patient from the first to the later time period, signifying an increased awareness of the risks of polypsychopharmacy. Other studies using AMSP data have found markedly lower rates of complex polypsychopharmacy for psychiatric inpatients with other severe mental disorders including schizophrenia (44.7%; Toto et al. 2019), major depressive disorder (45.7%; Seifert et al. 2021) and borderline personality disorder (54%; Bridler et al. 2015). So, while complex polypsychopharmacy is generally commonly encountered in psychiatric inpatients, it appears to be of particular significance in acute BD-DE.

Similar treatment patterns with complex multi-psychotropic drug regimens in patients with BD-DE have been reported by others (Goldberg et al. 2009; Greil et al. 2012; Golden et al. 2017; Haeberle et al. 2012), although polypsychopharmacy affects patients across all phases of BD (Fornaro et al. 2016; Golden et al. 2017), including maintenance therapy (Amerio et al. 2021). While previous AMSP studies indicated an increasing prevalence of triple and quadruple therapies and decreasing use of monotherapies in acute BD-DE from 1994–2009 (Greil et al. 2012; Haeberle et al. 2012), the present study revealed the opposite: We found a decrease in the average number of psychotropic drugs per patient from 2007–2010 to 2021–2024, most significantly for the use of four psychotropic drugs, as well as a significant increase in psychotropic monotherapy.

Several factors may be relevant contributors to the high prevalence of polypsychopharmacy in BD-DE. First of all, when experiencing an acute episode of BD, additional psychotropic drugs are often prescribed alongside the previously administered maintenance therapy (Kim et al. 2021). As BD-DE is particularly difficult to treat (Grande et al. 2016) and most drugs are of limited efficacy (Yildiz et al. 2023; Yalin et al. 2026), many patients experience incomplete remission. In general, the response rate to treatment with psychotropic drugs in BD-DE is 55.1% compared to 39.2% placebo response indicating a modest relative effect (relative risk = 1.29) and that a substantial proportion of patients do not achieve meaningful clinical response (Iovieno et al. 2016). These unmet therapeutic needs may contribute to the use of additional drugs, while at the same time the previously used – perhaps even ineffective – drugs are continued in fear of otherwise worsening symptoms (Kim et al. 2021). Moreover, treatment guidelines lack clear recommendations for when to discontinue drugs used to treat an acute episode of BD (Kim et al. 2021). In addition, comorbid symptoms such as psychotic features, insomnia and anxiety may prompt clinicians to prescribe adjunctive drugs, further contributing to an extensive medication list. Complex drug regimens may also be a reflection of higher illness severity – including the presence of psychotic features – and treatment resistance (Kim et al. 2021; Golden et al. 2017), therefore, a high rate of patients with complex polypsychopharmacy within this study’s inpatient setting can be expected. Risk factors for complex polypsychopharmacy include female sex, age > 50 years, lower dosages, comorbid mental disorders such as personality disorders and posttraumatic stress disorder, and a history of suicide attempts (Goldberg 2019). Although evidence suggesting that more complex treatment regimens yield better outcomes is lacking, polypsychopharmacy is to date one of the main contributors for the deviation from guideline recommendations (Kim et al. 2021; Goldberg 2019). Specifically in acute BD-DE, current guidelines discourage complex drug regimens and recommend limiting treatment to no more than two psychotropic drugs, typically consisting of combinations with mood stabilizing AED, SGA and lithium, while treatment with adjunctive ADD is considered second-line (Yatham et al. 2018). Among the most frequently implemented combination strategies found in the present study were those centered around the use of APD. The use of APD + ADD, which was found in more than half of all patients with BD-DE from 2007–2024, remained unchanged over time, indicating yet another deviation from guideline recommendations. We also found that nearly one fifth of patients with BD-DE received two or more ADD, a treatment strategy which is vastly understudied (Goldberg 2019). However, the use of two ADD significantly decreased over time in the present analysis.

While complex polypsychopharmacy appears to be prevalent in patients with BD, it is important to acknowledge the considerable risks and disadvantages associated with this treatment strategy. In addition to an increased risk of drug-drug interactions and adverse drug reactions (de Leon and Spina 2018), complex polypsychopharmacy has been associated with both poorer treatment outcomes, reduced adherence (Fung et al. 2019) and a higher risk of rehospitalization (Golden et al. 2017). Of note, poorer adherence and inferior treatment outcomes are of course closely related. Although this was not an outcome further analyzed in the present study, previous research has suggested that patients with BD-DE treated with lithium may be affected by (complex) polypsychopharmacy to a lesser extent than those without lithium (Goldberg et al. 2009; Greil et al. 2012; Golden et al. 2017). However, a recent study using AMSP data challenges this notion by indicating the opposite finding: Bipolar patients (all phases) with lithium receive significantly more psychotropic drugs than those without (3.4 vs 2.9, *p* < 0.01; Greil et al. 2025).

## Strengths and limitations

The present analysis uses data from the long-standing AMSP project which provides a standardized, multicentric framework for the systematic assessment of psychotropic drug use in routine clinical practice. This ensures a high level of methodological consistency and allows comparability with other AMSP studies. The analysis includes a large inpatient sample of 1,902 individuals over an 18-year period enabling the analysis of temporal trends in psychotropic drug use in a real-world setting.

Several limitations should be acknowledged. First, the data was obtained from reference-day surveys conducted in hospitals with consistent participation in the AMSP project from 2007–2024. While this approach improves longitudinal consistency and comparability, it may not adequately reflect drug use in other patient settings, other hospitals or other countries. Further, causal interpretations and conclusions regarding treatment duration, dosing, adherence, cross-titration strategies and clinical outcomes such as remission or relapse cannot be made. Other factors with relevance to drug selection such as patient preference as well as psychiatric and somatic comorbidities were not considered in this analysis. It is, for example, conceivable, that underlying comorbid anxiety disorders prompted the frequent use of ADD within this cohort. Moreover, drug use recorded on reference days may include drugs used temporarily for short-term symptom management or on an as-needed basis (e.g., low-dose quetiapine or mirtazapine to treat insomnia), therefore overestimating the prevalence of polypsychopharmacy. Information on non-pharmacological treatment strategies such as psychotherapy and neuromodulatory interventions (e.g., electroconvulsive therapy, repetitive transcranial magnetic stimulation) was not available.

## Conclusion and clinical implications

The present analysis indicates a substantial shift in the pharmacological treatment of acute BD-DE towards a higher utilization of SGA and a stable use of lithium, accompanied by a declining use of ADD and AED from 2007 to 2024. Despite a modest reduction in the average number of psychotropic drugs, complex polypsychopharmacy remains a highly prevalent treatment strategy in BD-DE. These findings suggest a discrepancy between guideline recommendations which discourage complex treatment regimens and real-world drug use patterns. However, these observations should be interpreted under consideration of this analysis’ observational design and absence of detailed clinical information that may have influenced drug choice. Deviations from guideline recommendations do not necessarily reflect a lack of interest or knowledge but may instead be indicative of the complexity of real-world clinical practice which requires treatment decisions extending beyond evidence derived from randomized controlled trials. Potential risks of complex treatment regimens regarding safety, treatment outcomes and adherence must be carefully and regularly assessed against the presumed advantages. Future research is warranted to better understand individual factors underlying real-world drug use patterns and their clinical outcomes in patients with complex mental illnesses. 

## Supplementary Information

Additional file 1.

## Data Availability

Data on an individual level generated and/or analyzed during the current study are not publicly available due to data protection regulations and ethical considerations. Summarized data on drug use relevant to the present study is provided in the tables of this manuscript.

## Abbreviations

ADD: Antidepressant drug
AED: Antiepileptic drug
AMSP: Drug safety in psychiatry (German: „Arzneimittelsicherheit in der Psychiatrie“)
BD: Bipolar disorder
BD-DE: Depressive episode in bipolar disorder
BD-ME: Manic episode in bipolar disorder
CI: Confidence interval
df: Degrees of freedom
DSM-V: Diagnostic and statistical manual of disease, 5th edition
FGA: First-generation antipsychotic
hp: High potency
HYPD: Hypnotic drug
ICD: International classification of disease
LI: Lithium
lp: Low potency
MAOI: Monoamine oxidase inhibitor
N: Number (of)
NaSSA: Noradrenergic and specific serotonergic antidepressant
OR: Odds ratio
PD: Psychotropic drug
PR: Prevalence ratio
SD: Standard deviation
SE: Standard error
SGA: Second-generation antipsychotic
SSNRI: Selective serotonin-norepinephrine reuptake inhibitor
SSRI: Selective serotonin reuptake inhibitor
TCA: Tricyclic antidepressant drug
TRD: Tranquilizing drug
