# Supplementary material for: Trajectories of psychotropic drug use patterns in psychiatric inpatients with acute depressive episodes of bipolar disorder: results from a pharmacovigilance program in German-speaking countries from 2007 to 2024
Source: Int J Bipolar Disord. 2026 Jul 31;14:28. doi: 10.1186/s40345-026-00435-9 (PMC13428732; doi:10.1186/s40345-026-00435-9)
Supplement: Supplementary file 1 — Additional file 1. [file 40345_2026_435_MOESM1_ESM.docx]

**Suppl. Table 1:** Number of psychotropic drugs used per patient and within drug classes in patients with acute depressive episodes in bipolar disorder from 2007–2024 and comparison between early (2007–2010) and late (2021–2024) study periods

|  | **2007–2024** | | **T1: 2007–2010** | | **T2: 2021–2024** | | **T2 vs T1** | |
| --- | --- | --- | --- | --- | --- | --- | --- | --- |
|  | **N patients** | **% of 1,902** | **N patients** | **% of 365** | **N patients** | **% of 321** | **PR (95% CI)** | **Welch's t-test, Cohen's *d*** |
| **Total** | 1,902 | **100.0%** | 365 | **100.0%** | **321** | **100.0%** |  |  |
| **N psychotropic drugs per patient** | | | | | | | | |
| **0** | 29 | 1.5% | 3 | 0.8% | 6 | 1.9% | 2.27 (0.57–9.02) |  |
| **1** | 165 | 8.7% | 21 | 5.8% | 38 | 11.8% | **2.06 (1.23–3.43)** |  |
| **2** | 450 | 23.7% | 71 | 19.5% | 77 | 24.0% | 1.23 (0.93–1.64) |  |
| **3** | 531 | 27.9% | 91 | 24.9% | 92 | 28.7% | 1.15 (0.90–1.47) |  |
| **4** | 384 | 20.2% | 92 | 25.2% | 50 | 15.6% | **0.62 (0.45–0.84)** |  |
| **≥ 5** | 343 | 18.0% | 87 | 23.8% | 58 | 18.1% | 0.76 (0.56–1.02) |  |
| **Mean ± SD** | 3.21 ± 1.55 | | 3.85 ± 1.69 | | 3.34 ± 1.75 | |  | t = -3.79, *p* < 0.001,  *d* = -0.30 |
| **Median** | 3 | | 3 | | 3 | |  |  |
| **N antipsychotic drugs per patient** | | | | | | | | |
| **0** | 453 | 23.8% | 109 | 29.9% | 62 | 19.3% | **0.65 (0.49–0.85)** |  |
| **1** | 1,018 | 53.5% | 199 | 54.5% | 158 | 49.2% | 0.90 (0.78–1.04) |  |
| **2** | 385 | 20.2% | 50 | 13.7% | 91 | 28.3% | **2.07 (1.52–2.82)** |  |
| **≥ 3** | 46 | 2.4% | 7 | 1.9% | 10 | 3.1% | 1.62 (0.63–4.22) |  |
| **Mean ± SD** | 1.02 ± 0.86 | | 1.05 ± 0.94 | | 1.22 ± 0.83 | |  | t = 2.68, *p* = 0.008,  *d* = 0.20 |
| **Median** | 1 | | 1 | | 1 | |  |  |
| **N antidepressant drugs per patient** | | | | | | | | |
| **0** | 565 | 29.7% | 80 | 21.9% | 103 | 32.1% | **1.48 (1.14–1.88)** |  |
| **1** | 992 | 52.2% | 195 | 53.4% | 169 | 52.6% | 0.99 (0.86–1.13) |  |
| **2** | 316 | 16.6% | 83 | 22.7% | 43 | 13.4% | **0.59 (0.42–0.82)** |  |
| **≥ 3** | 29 | 1.5% | 7 | 1.9% | 6 | 1.9% | 0.97 (0.33–2.87) |  |
| **Mean ± SD** | 0.84 ± 0.84 | | 1.05 ± 0.91 | | 0.89 ± 0.88 | |  | t = -2.36, *p* = 0.018,  *d* = -0.18 |
| **Median** | 1 | | 1 | | 1 | |  |  |
| **N antiepileptic drugs per patient** | | | | | | | | |
| **0** | 1,059 | 55.7% | 158 | 43.3% | 210 | 65.4% | **1.51 (1.31–1.74)** |  |
| **1** | 733 | 38.5% | 169 | 46.3% | 93 | 29.0% | **0.63 (0.51–0.77)** |  |
| **2** | 94 | 4.9% | 32 | 8.8% | 16 | 5.0% | 0.57 (0.32–1.02) |  |
| **≥ 3** | 16 | 0.8% | 6 | 1.6% | 2 | 0.6% | 0.38 (0.08–1.86) |  |
| **Mean ± SD** | 0.51 ± 0.65 | | 0.69 ± 0.72 | | 0.41 ± 0.62 | |  | t = -5.58, *p* < 0.001,  *d* = -0.42 |
| **Median** | 0 | | 1 | | 0 | |  |  |

PR printed in bold indicate a significant change. **N:** number (of); **PR:** prevalence ratio; **CI:** confidence interval; SD: standard deviation

**Suppl. Table 2:** Utilization of dual combinations of psychotropic drug classes with different psychotropic drug groups in patients with acute depressive episodes in bipolar disorder from 2007–2024 and comparison between early (T1: 2007–2010) and late (T2: 2021–2024) study periods

|  |  | **2007–2024** | | **T1: 2007–2010** | | **T2: 2021–2024** | | **T2 vs T1** |
| --- | --- | --- | --- | --- | --- | --- | --- | --- |
|  |  | **N** | **% of 1,902** | **N** | **% of 365** | **N** | **% of 321** | **PR (95% CI)** |
| **APD class** | | | | | | | | |
| SGA + | APD | 417 | 21.9% | 56 | 15.3% | 96 | 29.9% | **1.95 (1.45–2.61)** |
|  | ADD | 872 | 45.8% | 169 | 46.3% | 150 | 46.7% | 1.01 (0.86–1.19) |
|  | AED | 550 | 28.9% | 122 | 33.4% | 77 | 24.0% | **0.72 (0.56–0.91)** |
|  | LI | 377 | 19.8% | 71 | 19.5% | 68 | 21.2% | 1.09 (0.81–1.47) |
|  | TRD | 392 | 20.6% | 85 | 23.3% | 58 | 18.1% | 0.78 (0.58–1.05) |
|  | HYPD | 151 | 7.9% | 43 | 11.8% | 16 | 5.0% | **0.42 (0.24–0.74)** |
| **ADD class** | | | | | | | | |
| SSRI + | APD | 196 | 10.3% | 66 | 18.1% | 77 | 24.0% | 1.33 (0.99–1.78) |
|  | ADD | 154 | 8.1% | 39 | 10.7% | 22 | 6.9% | 0.64 (0.39–1.06) |
|  | AED | 231 | 12.1% | 61 | 16.7% | 39 | 12.1% | 0.73 (0.50–1.06) |
|  | LI | 157 | 8.3% | 34 | 9.3% | 31 | 9.7% | 1.04 (0.65–1.65) |
|  | TRD | 139 | 7.3% | 33 | 9.0% | 21 | 6.5% | 0.72 (0.43–1.22) |
|  | HYPD | 48 | 2.5% | 12 | 3.3% | 6 | 1.9% | 0.57 (0.22–1.50) |
| SSNRI + | APD | 339 | 17.8% | 69 | 18.9% | 51 | 15.9% | 0.84 (0.60–1.17) |
|  | ADD | 162 | 8.5% | 44 | 12.1% | 24 | 7.5% | 0.62 (0.39–1.00) |
|  | AED | 206 | 10.8% | 55 | 15.1% | 25 | 7.8% | **0.52 (0.33–0.81)** |
|  | LI | 138 | 7.3% | 25 | 6.8% | 24 | 7.5% | 1.09 (0.64–1.87) |
|  | TRD | 113 | 5.9% | 32 | 8.8% | 11 | 3.4% | **0.39 (0.20–0.76)** |
|  | HYPD | 57 | 3.0% | 19 | 5.2% | 6 | 1.9% | **0.36 (0.15–0.89)** |
| NaSSA + | APD | 198 | 10.4% | 43 | 11.8% | 25 | 7.8% | 0.66 (0.41–1.06) |
|  | ADD | 181 | 9.5% | 44 | 12.1% | 25 | 7.8% | 0.65 (0.40–1.03) |
|  | AED | 138 | 7.3% | 45 | 12.3% | 15 | 4.7% | **0.38 (0.22–0.67)** |
|  | LI | 94 | 4.9% | 26 | 7.1% | 17 | 5.3% | 0.74 (0.41–1.34) |
|  | TRD | 99 | 5.2% | 30 | 8.2% | 14 | 4.4% | **0.53 (0.29–0.98)** |
|  | HYPD | 42 | 2.2% | 20 | 5.5% | 4 | 1.2% | **0.23 (0.08–0.66)** |
| TCA + | APD | 96 | 5.0% | 28 | 7.7% | 11 | 3.4% | **0.45 (0.23–0.88)** |
|  | ADD | 71 | 3.7% | 18 | 4.9% | 9 | 2.8% | 0.57 (0.26–1.25) |
|  | AED | 59 | 3.1% | 22 | 6.0% | 6 | 1.9% | **0.31 (0.13–0.76)** |
|  | LI | 59 | 3.1% | 18 | 4.9% | 5 | 1.6% | **0.32 (0.12–0.84)** |
|  | TRD | 49 | 2.6% | 20 | 5.5% | 2 | 0.6% | **0.11 (0.03–0.48)** |
|  | HYPD | 16 | 0.8% | 7 | 1.9% | 1 | 0.3% | 0.16 (0.02–1.31) |

PR printed in **bold** indicate a significant change. **N:** number (of); **PR:** prevalence ratio; **CI:** confidence interval; **ADD:** antidepressant drug; **SSRI:** selective serotonin reuptake inhibitor; **SSNRI:** selective serotonin-norepinephrine reuptake inhibitor; **TCA:** tricyclic antidepressant; **NaSSA:** noradrenergic and specific serotonergic antidepressant; **MAOI:** monoamine oxidase inhibitor; **APD:** antipsychotic drug; **FGA:** first-generation antipsychotic drug; **lp:** low potency; **hp:** high potency; **SGA:** second-generation antipsychotic drug; **HYPD:** hypnotic drug; **TRD:** tranquilizing drug; **AED:** antiepileptic drug; **LI**: lithium
